# Macrophages in acne vulgaris: mediating phagocytosis, inflammation, scar formation, and therapeutic implications

**DOI:** 10.3389/fimmu.2024.1355455

**Published:** 2024-03-14

**Authors:** Yibo Feng, Jiaqi Li, Xiaohui Mo, Qiang Ju

**Affiliations:** Department of Dermatology, Renji Hospital, Shanghai Jiao Tong University School of Medicine, Shanghai, China

**Keywords:** acne vulgaris, macrophages, *Cutibacterium acnes*, inflammation, therapeutics

## Abstract

Macrophages serve as a pivotal nexus in the pathogenesis of acne vulgaris, orchestrating both the elimination of *Cutibacterium acnes* (*C. acnes*) and lipid metabolic regulation while also possessing the capacity to exacerbate inflammation and induce cutaneous scarring. Additionally, recent investigations underscore the therapeutic potential inherent in macrophage modulation and challenge current anti-inflammatory strategies for acne vulgaris. This review distills contemporary advances, specifically examining the dual roles of macrophages, underlying regulatory frameworks, and emergent therapeutic avenues. Such nuanced insights hold the promise of guiding future explorations into the molecular etiology of acne and the development of more efficacious treatment modalities.

## 1 Introduction

Acne vulgaris (AV) denotes a dermatological disorder exhibiting a high prevalence especially in adolescents, primarily impacting follicular sebaceous gland units (1, 2). In regions notably the face, chest, and back, enriched with sebaceous glands, the condition primarily presents as lesions characterized by open (blackheads) and closed (whiteheads) comedones, accompanied by additional dermatological features such as papules, pustules, and subsequent scarring (3). Although not life-threatening, the profound psychological ramifications stemming from acne due to its detriment to appearance and self-esteem are significant, encompassing anxiety, depression, and potential suicidal tendencies (4). The intricate pathogenesis of acne involves four key processes, including hyperkeratosis of the hair follicle, androgen-induced alterations in sebaceous products, *C. acnes* colonization, and associated inflammatory responses (5). While inflammatory events pervade AV, their precise immunological underpinnings warrant further investigation (6).

Macrophages, pivotal elements of the immune system, pervade skin tissues, orchestrating immune responses, inflammation regulation, and tissue regeneration (7). Macrophage polarization, coupled with the ensuing metabolic reprogramming, commands a pivotal position in contemporary immune regulation research. A confluence of studies elucidates the characterization of macrophages in disparate immune microenvironments, spotlighting seminal regulators of macrophage polarization and underscoring the instrumental role of metabolic reprogramming in orchestrating the activation toward pro-inflammatory and anti-tumor polarization (8, 9). While within dermatology, dysregulation of macrophage polarization significantly influences the pathogenesis of inflammatory skin diseases, such as psoriasis, Behçet’s disease, and rosacea (10–12).

Recent investigations delineate the multifaceted roles macrophages assume in acne formation: they contribute beneficially by clearing pathogens and regulating lipid metabolism, yet exert adverse impacts on inflammation and scarring. Despite converging evidence, strategies targeting macrophage regulation in acne vulgaris treatments remain varied. Delving deeper into the roles of macrophages in acne vulgaris is imperative for pioneering therapeutic interventions. This review elucidates recent insights into macrophage involvement in the pathogenesis of acne vulgaris, aiming to foster further research and therapeutic advancements.

## 2 The role of macrophages in cutaneous tissues

Skin macrophages are classified into tissue-resident and infiltrating types. Tissue-resident macrophages, originating from precursor cells during embryogenesis, are maintained through adulthood via self-renewal (13). Conversely, infiltrating macrophages differentiate from monocytes that, originating from bone marrow, infiltrate tissues in response to stimuli such as Macrophage Colony-Stimulating Factor (M-CSF), Granulocyte-Macrophage Colony-Stimulating Factor (GM-CSF), Interleukin (IL)-4, IL-10, and IL-13.

In skin, macrophages play crucial roles in maintaining homeostasis and mediating immune responses. Under normal conditions, they sustain tissue homeostasis by clearing cellular debris and secreting cytokines (14). In sebum-rich areas, they maintain lipid equilibrium by phagocytosing sebum (15). Upon injury, they modulate inflammatory responses and mediate adaptive immunity by presenting antigens to T cells (16). In later stages, macrophages secrete growth factors and cytokines to promote cell proliferation and matrix remodeling, aiding wound healing.

**Figure 1:**
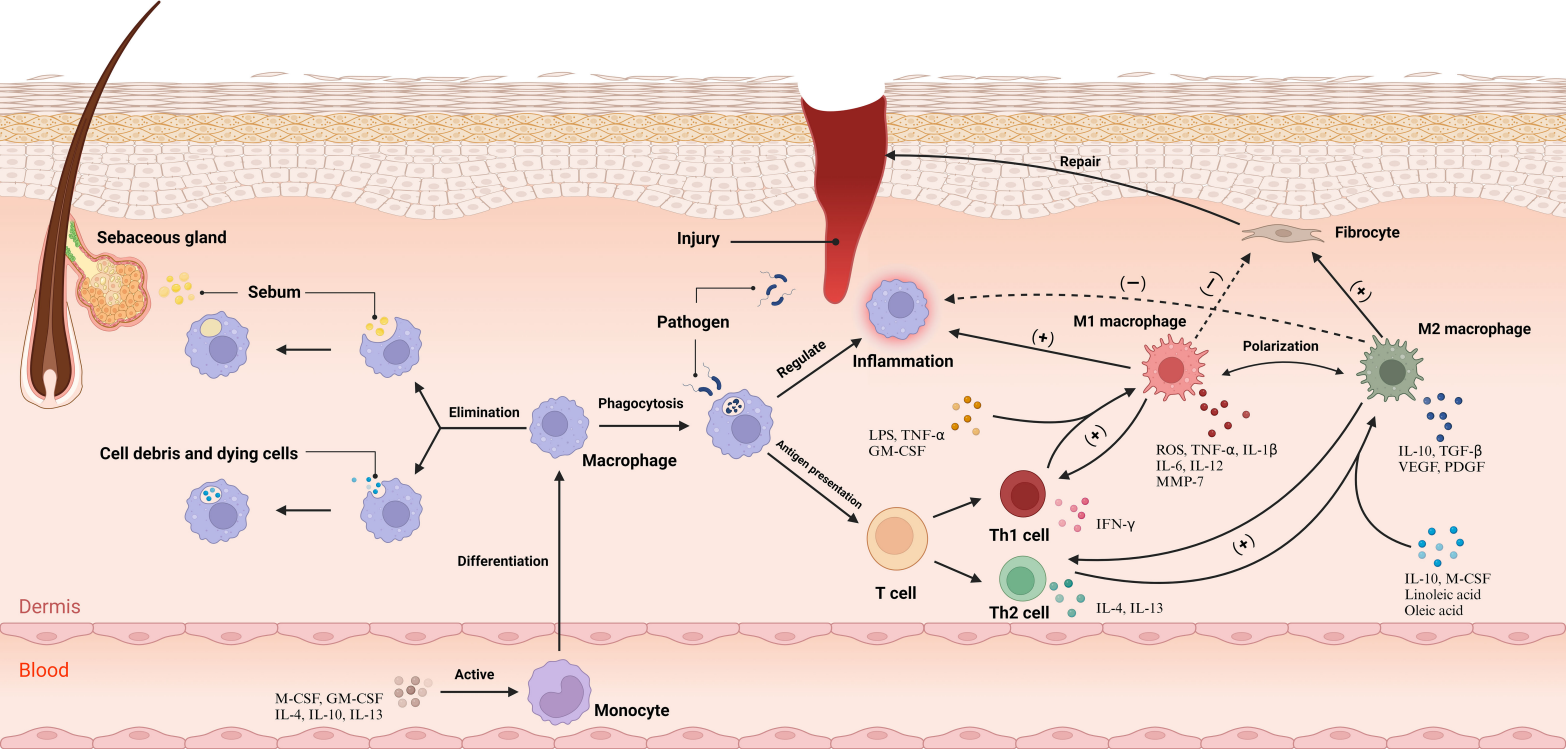
Under physiological conditions, macrophages play pivotal roles, namely the clearance of cellular remnants and apoptotic cells, phagocytosis of lipids, and the sustenance of dermal tissue equilibrium. Upon the manifestation of a wound, Cytokines incite monocytes within the bloodstream, catalyzing their differentiation into macrophages, which subsequently permeate the dermal tissue. Macrophages undertake the phagocytosis of pathogens and orchestrate the inflammatory responses while mediating antigen presentation to T cells. This interaction induces the differentiation of T cells into Th1 and Th2 subsets. Subsequently, Th1 cells augment M1 polarization, and Th2 cells enhance M2 polarization. M1 macrophages primarily emit pro-inflammatory cytokines and attenuate fibroblast function, while M2 macrophages release cytokines that mitigate inflammation and elevate fibroblast activity, promoting tissue repair. The cytokines secreted by M1 and M2 macrophage subtypes stimulate Th1 and Th2 cells, respectively, maintaining a balanced immune response within the dermal ecosystem. CCL6, chemokine (C-C motif) ligand 6; GM-CSF, Granulocyte-Macrophage Colony-Stimulating Factor; IFN-γ, Interferon-gamma; IL-1, Interleukin-1; LPS, Lipopolysaccharide; M-CSF, Macrophage Colony-Stimulating Factor; MMP-7, Matrix Metallopeptidase 7; PDGF, Platelet-Derived Growth Factor; ROS, reactive oxygen species; TGF-β, Transforming Growth Factor-beta; Th1, T helper 1 cell; TNF-α, Tumor Necrosis Factor-alpha; VEGF, Vascular Endothelial Growth Factor. Adapted from “Skin (Layout)” by BioRender.com (2023). Retrieved from https://app.biorender.com/biorender-templates.

Macrophage polarization is pivotal for their role in skin. Lipopolysaccharide (LPS), GM-CSF, Tumor Necrosis Factor (TNF)-α, and Interferon (IFN)-γ produced by Th1 cells mediate the classical activation pathway, polarizing macrophages to M1 type, which produce high levels of reactive oxygen species (ROS) and pro-inflammatory cytokines like TNF-α, IL-1β, IL-6, and IL-12, enhancing inflammation and eliminating pathogens (17). Conversely, chemokine (C-C motif) ligand 6 (CCL6), M-CSF, linoleic acid, oleic acid, IL-4, and IL-13 produced by Th2 cells mediate the alternative activation pathway, polarizing to M2 type (18). M2 macrophages suppress the inflammatory response by producing IL-10. They further promote fibroblast proliferation, collagen synthesis, and angiogenesis through the release of Transforming Growth Factor (TGF)-β, Platelet-Derived Growth Factor (PDGF), and Vascular Endothelial Growth Factor (VEGF), facilitating tissue repair (19). In contrast, Matrix Metalloproteinases (MMPs) expressed by M1 macrophages degrade collagen, inhibiting fiber formation. IL-12 and IL-10, in turn, stimulate the differentiation of T cells to Th1 and Th2 cells, respectively (20) (**Figure 1**). Comprehensively, M1 macrophages within the dermal ecosystem play a pivotal role in instigating inflammatory responses that not only target pathogens but also contribute to the resultant tissue damage. Simultaneously, M2 macrophages, in their pivotal role, regulate and suppress excessive inflammatory reactions to forestall damage, also facilitating tissue repair and wound healing. The interplay between shifts in the cytokine milieu and macrophage plasticity enables a flexible modulation of M1 and M2 functionalities, a cornerstone for preserving systemic equilibrium. Disruption of this equilibrium heralds pathological sequelae and the inception of disease states. For instance, single-cell RNA sequencing insights from hidradenitis suppurativa illustrate that the STAT1/IFN axis propels macrophages towards an M1-skewed pro-inflammatory phenotype, exacerbating inflammation and promoting immune cell ingress (21). Conversely, the predilection for M2 macrophage polarization not only underpins tumorigenesis but also catalyzes fibrosis and granuloma evolution, potentially correlating with the systemic manifestations observed in sarcoidosis (22, 23).

## 3 The central role of inflammation in the pathogenesis of acne vulgaris

The onset of acne lesions is characterized by the emergence of microcomedones, consequent to hyperkeratinization occurring within the funnel section of the follicular canal, causing pore obstruction and the subsequent development of comedones (24). Upon exposure to air, blackheads undergoes oxidation and darkening, contrasting with whiteheads, which remains subcutaneous, manifesting a white or normal-skinned appearance. *C. acnes* multiply within these obstructed follicles, inciting an inflammatory response manifested by redness, swelling, and pus (25). Without adequate intervention, such lesions may intensify into painful pustules, cysts, or nodules, risking irreversible damage and scarring in the absence of proper treatment or when exacerbated.

The manifestation of acne lesions is precipitated by the synergistic interaction of four predominant causative factors (26). The upsurge in sebum production and augmented epithelial keratinization within hair follicles, instigated by abnormalities in androgen metabolism, cultivate an environment optimal for the prolific expansion of *C. acnes*. Metabolites of *C. acnes* incite inflammation within follicular and perifollicular regions, coupled with an ensuing immune cell infiltration of the follicular wall, which may culminate in follicular rupture. Subsequent to this, the materials within pimples permeate the dermal tissue, escalating the inflammation and thereby engendering pustules, granulomas, and symptoms indicative of systemic inflammation.

Inflammatory reactions are omnipresent throughout acne’s progression. Research conducted by Jeremy et al. has revealed prominent escalations in CD3+ and CD4+ T cells, macrophages, and IL-1α in the dermis surrounding unaltered hair follicles in individuals afflicted with acne, suggesting inflammation as a precursor to the manifestation of acne (6). Importantly, IL-1α is capable of activating basal keratinocytes through autocrine production, prompting the augmented proliferation of suprabasal cells and subsequently inducing hyperkeratosis in the follicular funnel (27). Subsequently, *C. acnes* facilitates the release of inflammatory cytokines, including TNF-α, IL-6, and IL-8 from dendritic cells and keratinocytes, initiating an extensive adaptive immune response (28). This series of events leads to the mobilization of macrophages and lymphocytes to the site, enhancing perifollicular cell infiltration due to elevated cytokine release, resulting in the formation of inflamed papules. Post follicular destruction, components such as keratin and hair elements incite severe inflammatory reactions, culminating in the development of pustules or granulomas. A significant correlation is observed between the severity and duration of inflammation and the inception of scars, as demonstrated by Carlavan et al., attributed to delayed inflammatory reactions enhancing the susceptibility to scarring in acne patients due to the dysregulation of the innate immune response (29). This investigation further identified elevated expression of various immunoglobulin genes and the pervasive infiltration of mature B cells within the long-lasting lesions of scarring patients. Concurrently, Holland and colleagues documented that lesions in scarring individuals exhibit diminished HLA-DR expression alongside sparse CD4+ T cell infiltration (30). Collectively, these observations underscore the potential role of adaptive immunity dysregulation in the etiology of acne scar formation.

## 4 Macrophages in acne: a dual facet in pathogenesis

Macrophages embody a paradoxical role in acne development, serving as both sentinels and provocateurs. Their vital functions include the regulation of lipid concentrations and facilitating the elimination of *C. acnes*. However, an immoderate immune reaction can provoke inflammation and subsequent acne scarring. It is imperative to comprehend their intricate roles to maintain physiological equilibrium and circumvent adverse pathological outcomes.

### 4.1 Macrophages and lipid metabolism regulation

Individuals with AV often exhibit abnormal plasma lipid profiles, characterized by elevated plasma lipoproteins and a decrease in High-Density Lipoprotein (HDL) cholesterol levels, particularly in severe acne conditions (31). These abnormalities in serum total cholesterol levels act as a substrate for androgen synthesis by the adrenal glands and gonads, intensifying the progression of AV through the increase of androgens (32).

In the context of the aforementioned lipid metabolism disruption, macrophages are crucial regulators, orchestrating the assimilation of oxidized low-density lipoprotein (LDL) and cholesterol. Macrophage scavenger receptor 1 (MSR1) facilitates the incorporation and breakdown of modified LDL, leading to intracellular cholesterol accumulation and influencing macrophage functionality (33). AbdElneam et al. found that of the four common haplotypes of the MSR1 gene, the most common haplotypes in patients with AV are TCAC and CAGG, whereas the most common haplotypes in healthy populations are TAAC and CCAC (34). Additionally, they observed a linkage disequilibrium in the MSR1 gene, indicating that genetic elements might significantly influence the development of AV by altering macrophage-mediated lipid regulation.

### 4.2 Engulfment and clearance of *C. acnes* by macrophages

Macrophage phagocytosis in acne lesions is prominently orchestrated by *C. acnes*, a Gram-positive, parthenogenetic anaerobic bacillus. This bacterium is pivotal, predominantly inhabiting the sebaceous glands of hair follicles in acne patients and playing a vital role in acne pathogenesis (35). It triggers the expression of IL-15 and GM-CSF via Toll-like receptor 2 (TLR2), fostering the differentiation of monocytes into CD209+ macrophages and CD1b+ dendritic cells (36). Comparatively, CD209+ macrophages exhibit superior efficacy in phagocytosing and curtailing the growth of *C. acnes*, becoming a major line of host defense against this bacterium.

Detailed analysis of skin biopsies from inflammatory acne lesions reveals macrophages’ ability to recognize and phagocytose *C. acnes* strains, with sebum playing a role in enhancing this ability (15). These macrophages utilize a variety of enzymes, such as lipoxygenase and myeloperoxidase, to produce antimicrobial agents ROS and reactive nitrogen intermediates (RNI) in lysosomes. These agents then fuse with phagosomes to eliminate the pathogens using ROS and RNI (37).

Notably, Do et al. found that excess squalene production by keratin-forming cells in acne lesions scavenges ROS (38). This also leads to a reduction in the expression of genes encoding oxidative enzymes produced by ROS and RNI, including the NADPH oxidase genes NOX1, NOS2, and NFKB. Furthermore, the expression of genes encoding ROS-induced oxidative pathway enzymes, such as the mitogen-activated protein kinase genes MAPK8, MAPK14, and NFKB, is also reduced. This renders TREM2 macrophages virtually devoid of antimicrobial activity, blocking the antimicrobial response and increasing the bacterial load. Consequently, this leads to the secretion of IL-18 and upregulation of inflammatory chemokine expression, instigating a disease site-specific inflammatory response. Remarkably, this phenomenon elucidates the efficacy of benzoyl peroxide in the clinical management of acne through the peroxidative destruction of *C. acnes*. Remarkably, this study illuminates an additional mechanism by which topical benzoyl peroxide combats acne, beyond its direct antibacterial action against *C. acnes*, anti-inflammatory effects, and comedolytic properties (4). It achieves this by generating oxygen radicals that negate squalene’s capacity to scavenge oxygen radicals, thereby countering the suppression of squalene’s antimicrobial potency on TREM2 macrophages.

**Figure 2:**
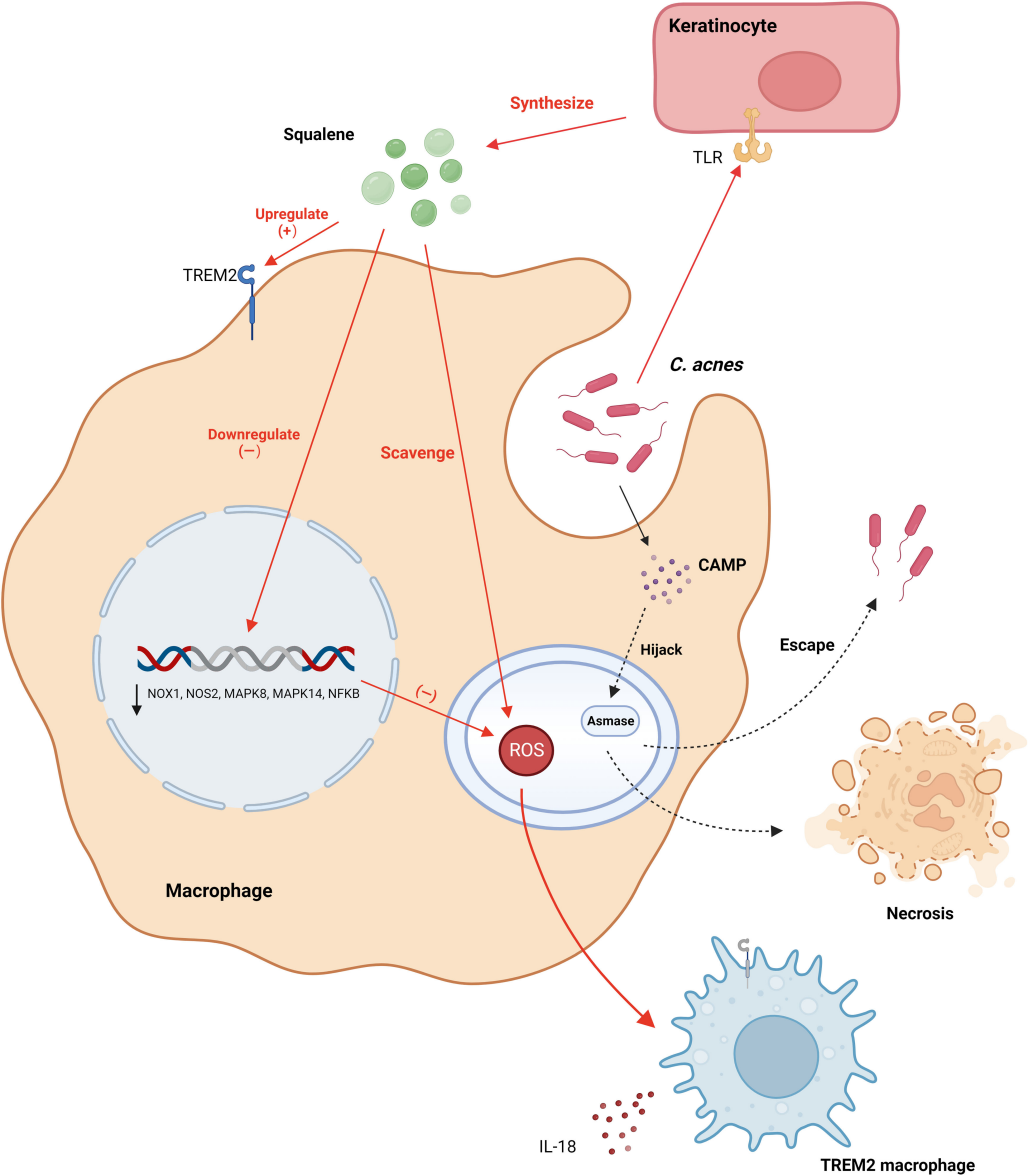
*C. acnes* modulates the secretion of squalene from keratinocyte cells via TLR receptors, elevating the expression of TREM2 receptors. This secretion mitigates reactive oxygen species within lysosomes, leading to the downregulation of genes encoding oxidative and pathway enzymes responsible for the production of ROS and RNI. Consequently, this leads to the transformation of macrophages into TREM2 macrophages, which primarily release pro-inflammatory factors and exhibit diminished proficiency in eliminating *C. acnes*. Additionally, *C. acnes* synthesizes the CAMP factor, which, facilitated by Asmase, either evacuates from macrophages or provokes macrophage necrosis. CAMP, Christie, Atkins, Munch-Peterson; TLR, Toll-Like Receptor; TREM2, Triggering Receptor Expressed on Myeloid cells 2. Created with BioRender.com.

Nakatsuji et al. elucidated that the synergistic interaction of the Christie, Atkins, Munch-Peterson (CAMP) factor from *C. acnes* and acidic sphingomyelinase (ASMase) from the host cells triggers an inflammatory response and inflicts cytotoxicity to macrophages (39). Based on these findings, Nakatsuji formulated two plausible hypotheses: *C. acnes* may evade the immune response by utilizing host ASMase, or the CAMP factors might manipulate host ASMase, thereby amplifying bacterial virulence, undermining macrophages, and obstructing the efficient clearance of *C. acnes* (**Figure 2**).

### 4.3 Macrophage-mediated inflammatory regulation in acne lesions

Macrophages exhibit a central role in mediating inflammatory responses within acne, governed by diversified pathways.

Sebaceous gland-originating lipids are crucial modulators of macrophage activities. A study led by Lovászi et al. elucidated that sebum is imperative for IL-1β secretion in *C. acnes*-activated macrophages, where components like oleic acid and squalene intensify IL-1β secretion (15). Intriguingly, oleic acid selectively moderates the expression of IL-6 and TNF-α, introducing a paradox considering its role in promoting M2 polarization and IL-1β secretion amplification. In contrast, linoleic acid exerts anti-inflammatory properties, reducing the secretion of prominent inflammatory mediators. On a different note, Tang et al.’s study provides insights into the synergistic secretion of IL-1β and TNF-α by sebaceous cell glands and macrophages in acne conditions (40). Sebaceous cell glands induce macrophage polarization to the M1 phenotype, exacerbating inflammatory reactions. Cannabidiol (CBD) is recognized to mitigate this interplay effectively.

Furthermore, keratinocytes residing in acne lesions orchestrate macrophage inflammatory responses. Research by Graham highlighted that *C. acnes* and its GroEL protein trigger keratinocytes to release IL-1α, Chemokine (C-X-C motif) Ligand 8 (CXCL8), GM-CSF, TNF-α, and human β-defensin-2 (hBD-2) via the TLR, enhancing macrophage activation and recruitment and intensifying inflammation (41, 42). Additionally, squalene, produced by keratinocytes, prompts macrophages to increase TREM2 receptor expression, amplifying inflammatory gene expression (38).

*C. acnes* plays a cardinal role in inciting the production of pro-inflammatory cytokines such as IL-1β, largely by the direct activation of Pattern Recognition Receptors (PRR) in macrophages, occurring primarily through Pathogen-Associated Molecular Patterns (PAMP) (43). This encompasses entities like TLR and Nod-like receptors (NLR). Tsai et al. delineated that *C. acnes* engages the MAPK and NF-κB signaling cascades via the TLR2 receptor on macrophages, thereby modulating the expression of inducible Nitric Oxide Synthase (iNOS)/NO and Cyclooxygenase-2 (COX-2)/Prostaglandin E2 (PGE2) (44). This interaction is reliant upon the nuanced phosphorylation of protein kinases JNK and EPK along with ROS-enhanced IKK protein phosphorylation, cumulatively activating AP-1 and NF-κB and consequently augmenting the production of NO and PGE2. Fischer and colleagues delineated that the activation of *C. acnes* is dependent on the junctional protein TIR-domain-containing adapter-inducing interferon-β (TRIF), initializing the cGAS-STING pathway and inducing the type I interferon axis in macrophages, catalyzing additional signaling pathways, including the JAK-STAT pathway, and modulating the inflammatory response (45). TRIF, predominantly a downstream of TLR3/4, does not ordinarily recognize Gram-positive bacteria. Fischer posited that TRIF signaling is coupled with TLR2, aiding macrophages in the activation of the IFN-I axis after the recognition of *C. acnes*. It’s worth noting that TLR4 also discerns Gram-positive bacteria, and, as previously highlighted, *C. acnes* can stimulate keratinocytes via both TLR2 and TLR4 (41, 42, 46). Consequently, the intricate mechanisms by which *C. acnes* activates the cGAS-STING pathway via TRIF remain to be explicated comprehensively.

**Figure 3:**
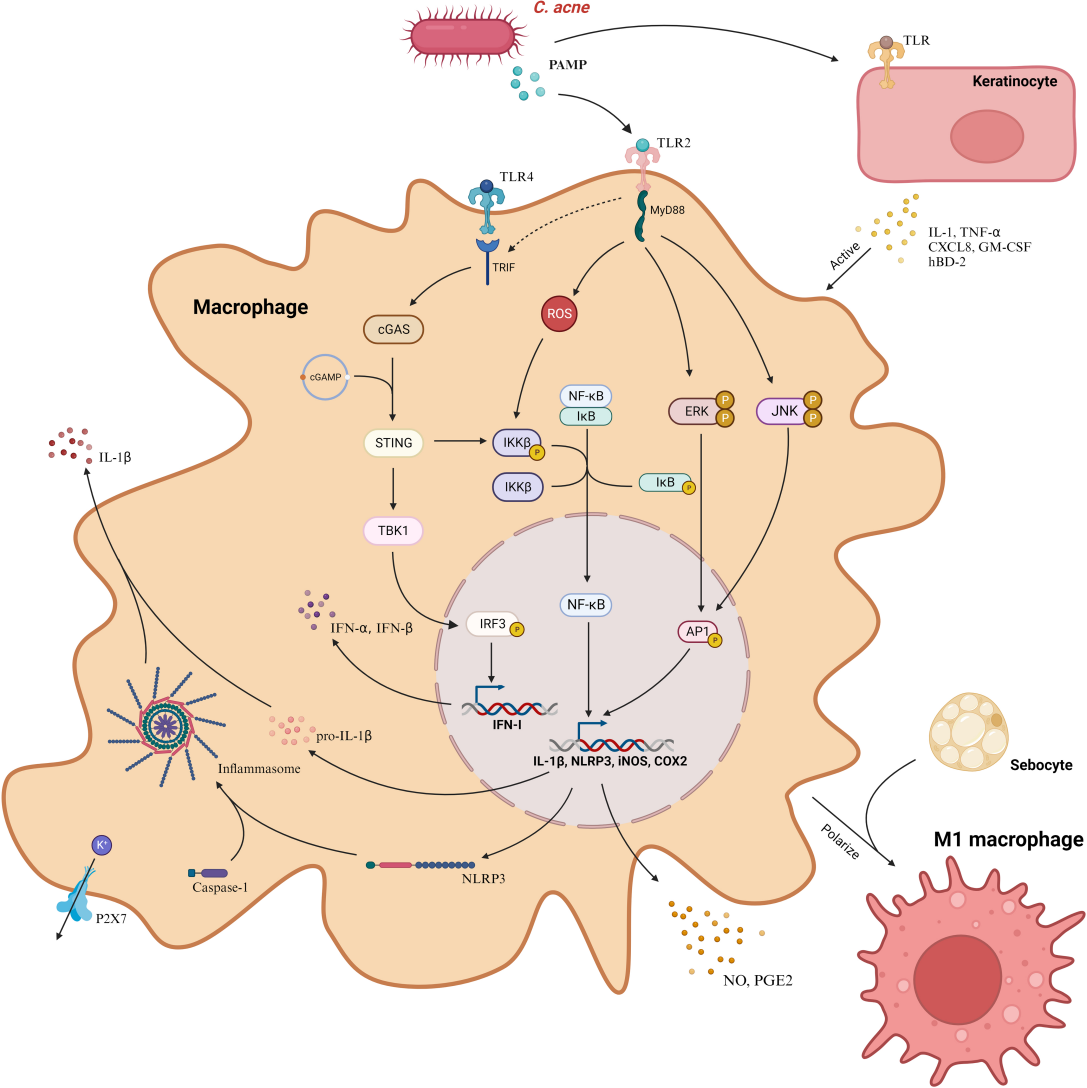
*C. acnes* predominantly instigates three distinctive signaling cascades in macrophages through PAMP, mediated via TLR2. These cascades encompass the ERK, JNK, and NF-κB pathways, which amplify the transcription of iNOS and COX2 genes, facilitating the liberation of NO and PGE2. Concurrently, *C. acnes* incites the cGAS-STING signaling cascade via TRIF, initiating the type I interferon axis. This bacterium also enhances the expression of NLRP3, which collaborates with caspase-1 to form the inflammasome. This assembly, reliant on K+ efflux, processes pro-IL-1β into IL-1β, enabling its release from the cell. Additionally, *C. acnes* induces keratinocytes to discharge cytokines, in turn, activating macrophages. Furthermore, in the presence of *C. acnes*, sebocytes facilitate the polarization of macrophages to the M1 phenotype. COX2, Cyclooxygenase-2; CXCL8, Chemokine (C-X-C motif) ligand 8; GM-CSF, Granulocyte-Macrophage Colony-Stimulating Factor; hBD-2, Human Beta-Defensin 2; IFN-I, Interferon Type I; IL-1β, Interleukin-1 beta; iNOS, Inducible Nitric Oxide Synthase; MyD88, Myeloid Differentiation Primary Response Protein 88; NLRP3, NOD-like Receptor 3; P2X7, Purinergic Receptor P2X7; PAMP, Pathogen-Associated Molecular Pattern; PGE2, Prostaglandin E2; TLR, Toll-Like Receptor; TNF-α, Tumor Necrosis Factor-alpha; TRIF, TIR-Domain-Containing Adapter-Inducing Interferon-β Protein. Created with BioRender.com.

*C. acnes* intricately modulates IL-1β secretion through NOD-like receptor family, pyrin domain-containing 3 (NLRP3), amplifying the transcription of both IL-1β and NLRP3 genes predominantly via the NF-κB pathway (47). Once transcribed within macrophages, NLRP3 emerges as a pivotal sensor, converging with the effector cysteine protease (caspase)-1 to orchestrate the inflammasome, thereby catalyzing the metamorphosis of pro-IL-1β to matured IL-1β for ensuing release (48). This mechanism, elucidated by Qin et al., is contingent upon K+ efflux (**Figure 3**). Regrettably, the absence of subsequent comprehensive studies renders this field ripe for further exploration. Indeed, inflammasomes are also capable of processing Gasdermin D (GSDMD), integrating it into the plasma membrane and creating pore structures to release pro-inflammatory cytokines, ultimately inducing cellular pyroptosis——a distinct, inflammation-associated form of programmed cell death instrumental in host defense and implicated in anti-tumor activities in certain cancers (47). However, in dermatological conditions, it predominantly manifests as a pathogenetic mechanism. Deng et al. delineated how SpeB, an exotoxin of Streptococcus pyogenes, triggers cutaneous keratinocyte pyroptosis through the cleavage of GSDMA (49). While Lian et al. highlighted the role of GSDMD-mediated keratinocyte pyroptosis in fostering hyperproliferation and aberrant differentiation, contributing to the development of psoriasis (50). Hence, probing the potential induction of macrophage cell pyroptosis by *C. acnes* is pivotal and promises profound insights into the pathogenesis of acne vulgaris.

Finally, genetic elements play a significant role in shaping the manner in which macrophages regulate inflammation. Resistin, a protein primarily secreted by macrophages, acts to elevate the expression of inflammatory mediators (51). A significant revelation made by Akcılar et al. was the identification of a protective association linked to the CG genotype of the resistin gene, correlating prominently with a decreased risk of acne vulgaris.

### 4.4 Influence of macrophages on the progression of acne scarring

Primary lesions in acne vulgaris frequently culminate in scarring, which, by altering physical appearance, can engender significant social and psychological ramifications (52). The genesis of scarring is multifaceted, implicating not only the intensity and duration of inflammation but also the intricate interplay of cytokines secreted by diverse macrophage subtypes, orchestrating dermal fibroblasts’ activity (53). Disruption in the equilibrium of pro- and anti-fibrinogen cytokines and growth factors precipitates aberrant extracellular matrix protein synthesis and compromises fibroblast functionality, culminating in wound remodeling, contraction, and subsequent scar manifestation (54).

Holland et al. elucidated disparate macrophage trajectories between not-scar-prone (NSP) and scar-prone (SP) patients (30). In NSP patients, pervasive macrophage infiltration coupled with augmented HLA-DR expression marked efficient antigen clearance and cellular activation, reverting to basal levels as lesions resolved. In contrast, SP patients evidenced sustained macrophage elevations and initial subdued, but progressively accentuated, HLA-DR expressions, indicating an escalating, specialized immune response, fostering angiogenesis and exacerbated inflammation, and culminating in scarring. Complementing this, Carlavan et al. detected enduring macrophage recruitment and irreversible glandular disruptions in SP patients, implying an elongated inflammatory state (29).

Macrophage polarization may further play a pivotal role in the pathogenesis of acne scarring. Under normal conditions, M1 macrophages predominantly clear pathogens and debris during wound healing’s initial phase. As healing advances to the proliferative phase, a critical shift to M2 macrophages occurs, promoting tissue regeneration and reducing inflammation. In the remodeling phase, M2 macrophages regulate collagen, essential for tissue restoration (55). Saint-Jean et al. discerned elevated IL-2 and IL-10 and attenuated MMP-9 proteins in SP patients, implicating a predominance of M2 macrophages (56). Besides, relationship between macrophage polarization and acne scarring still lacks more empirical elucidation, but there have been many solid proofs for other scarring phenomena. It is imperative to acknowledge that a heightened prevalence of pre-injury M2 cells, a suppression of M1 expression in preliminary phases, and a subsequent deferred M2 expression in terminal phases, are associated with the evolution of pathological scarring (57). The accentuated presence of M2 cells along scar peripheries and within the superficial dermis underscores the potential ramifications of macrophage hyperactivity in disrupting the equilibrium of collagen synthesis and catabolism, thereby precipitating hyperfibrosis (58, 59). This scenario is analogous to the complexities observed in numerous chronic wounds, wherein the activation of M1 macrophages is inhibited, preventing a full transition to the M2 phenotype and thus arresting the repair mechanism within the inflammatory stage (60). Recent investigations have illuminated that SPP1+ macrophages exhibiting an M2 macrophage phenotype, in conjunction with POSTN+ fibroblasts, precipitate the development of fibrotic scars in acne keloidalis through interactions mediated by the SPP1 axis (61).

## 5 Therapeutic strategies and potential approaches addressing macrophages in acne vulgaris

The comprehensive understanding of the pathogenesis of acne vulgaris is yet to be fully attained. Nonetheless, numerous empirical studies have highlighted the ability of existing therapeutic strategies to alleviate acne by modulating macrophage functionality. Furthermore, multiple approaches, which focus specifically on macrophages, display considerable potential for therapeutic advancement in acne vulgaris, signaling new directions in research and application. Intriguingly, our scrutiny of acne treatment strategies targeting macrophages uncovered a general trend towards dampening inflammatory signaling pathways and cytokine production, with strategies encompassing M2 macrophage polarization among others. Remarkably, photodynamic therapy (PDT), used to treat severe acne, delineates an antithetical mechanism by advocating for M1 macrophage polarization, thereby escalating inflammation (62). This tiered therapeutic paradigm for acne articulates a groundbreaking perspective: advocating pro-inflammatory interventions for individuals grappling with severe acne, in stark divergence from the anti-inflammatory modalities prescribed for mild conditions. The discrepancy between fostering and mitigating inflammation unmistakably signals the presence of an alternative to current therapeutic modalities, signaling a departure from established treatments and underscoring the pressing need for further investigation to validate these novel approaches.

### 5.1 Retinoids

Retinoids, esteemed in dermatological domains, are quintessential in acne vulgaris therapy, administered both topically and orally. They exemplify diverse pharmacological competence, optimizing follicular keratinization, suppressing the proliferation of *C. acnes*, exhibiting anti-inflammatory traits, and forestalling scarring (63). Notably, All-trans retinoic acid (ATRA) and adapalene (AD) are paramount in this pharmacological class.

Retinoids enhance macrophage phagocytosis and clearance via the inhibition of squalene sebum (38). Liu et al. elucidated that ATRA not only moderates the expression of TLR2, attenuating C. acnes-induced inflammatory cytokine production, but also orchestrates the differentiation of monocytes into macrophages over dendritic cells, intensifying the phagocytosis of C. acnes and curtailing its proliferation (64). Further, Ji et al. validated the therapeutic efficacy of the electrostatically optimized adapalene-loaded emulsion, illustrating its significant inhibitory impact on the macrophage expression of pro-inflammatory substances (65).

### 5.2 Natural product drugs

Natural entities, including specific plant and animal extracts, have long been integral to acne treatment methodologies, predominantly as ethnomedicines, across diverse cultures. Several of these derivatives are renowned for their anti-inflammatory capacities, with a focus on modulating signaling pathways in macrophages (**Table 1**).

**Table 1:** Natural product drugs employed for Acne Vulgaris intervention by addressing action on macrophages.

Chemical	Description	Mechanism of action	Source	References
EAAD	Ethyl acetate extract from Angelica Dahuricae Radix	Inhibition of inflammatory responses through suppression of the NF-κB and MAPK pathways	Angelica dahurica	(66)
Quercetin	Plant polyphenolic of flavonoid	Inhibition of inflammatory responses through suppression of the MAPK pathway	Fruits, leaves, and vegetables	(67)
Cembrene Diterpenoids	Obtained from Soft Coral Sinularia flexibilis	Inhibition of inflammatory responses through suppression of the MAPK pathway	*S. flexibilis*	(68)
Apigenin	Phenolic compound	Inhibition of inflammatory responses through suppression of the MAPK pathway	Fruits, leaves, and vegetables	(69)
Kaempferia parviflora Extract	Plant Extract	Inhibition of inflammatory responses through suppression of the NF-κB pathway	*Kaempferia parviflora*	(70)
licochalcone A	Chalconoid isolated from the root of Glycyrrhiza inflate	Inhibition of inflammatory responses through suppression of NLRP3 inflammasome activation	*Glycyrrhiza inflate*	(71)
Jumihaidokuto(JHT)	Pharmaceutical-grade traditional medicine	Promotion of inflammatory responses through induction of M1 polarization	Japanese medicine	(72)

Kang et al. elucidated that ethyl acetate extract from *Angelica Dahuricae* Radix (EAAD) effectively inhibits iNOS and COX-2 expression and concurrently reduces the production of NO, PGE2, and TNF-α in macrophages by blocking the NF-κB and MAPK pathways (66). Analogously, substances such as quercetin, apigenin, and cembrene diterpenoids from the cultured soft coral *Sinularia flexibilis* inhibit the MAPK pathway in macrophages, thereby alleviating inflammation (67–69). On the other hand, *Kaempferia parviflora* extract and licochalcone A demonstrate anti-inflammatory effects by obstructing the NF-κB pathway and NLRP3 inflammasomes respectively (70, 71). In a distinctive study, Sekiguchi et al. revealed that JHT, a conventional Japanese medicine for acne, promotes the differentiation of monocytes to M1-type macrophages and intensifies macrophage infiltration, suggesting a potential improvement in acne symptoms through the enhancement of M1-type macrophage functions (72).

### 5.3 Photodynamic therapy

5-Aminolevulinic Acid Photodynamic Therapy (ALA-PDT) is emerging as a groundbreaking therapeutic approach for acne vulgaris, distinguished by its unequivocal efficacy, minimal invasiveness, and lack of systemic side effects (73). Post-ALA-PDT, a significant initial symptom is a severe inflammatory response, evidenced by erythema and pustules, with the intensity of the response being directly proportional to the effectiveness of the treatment (74).

**Figure 4:**
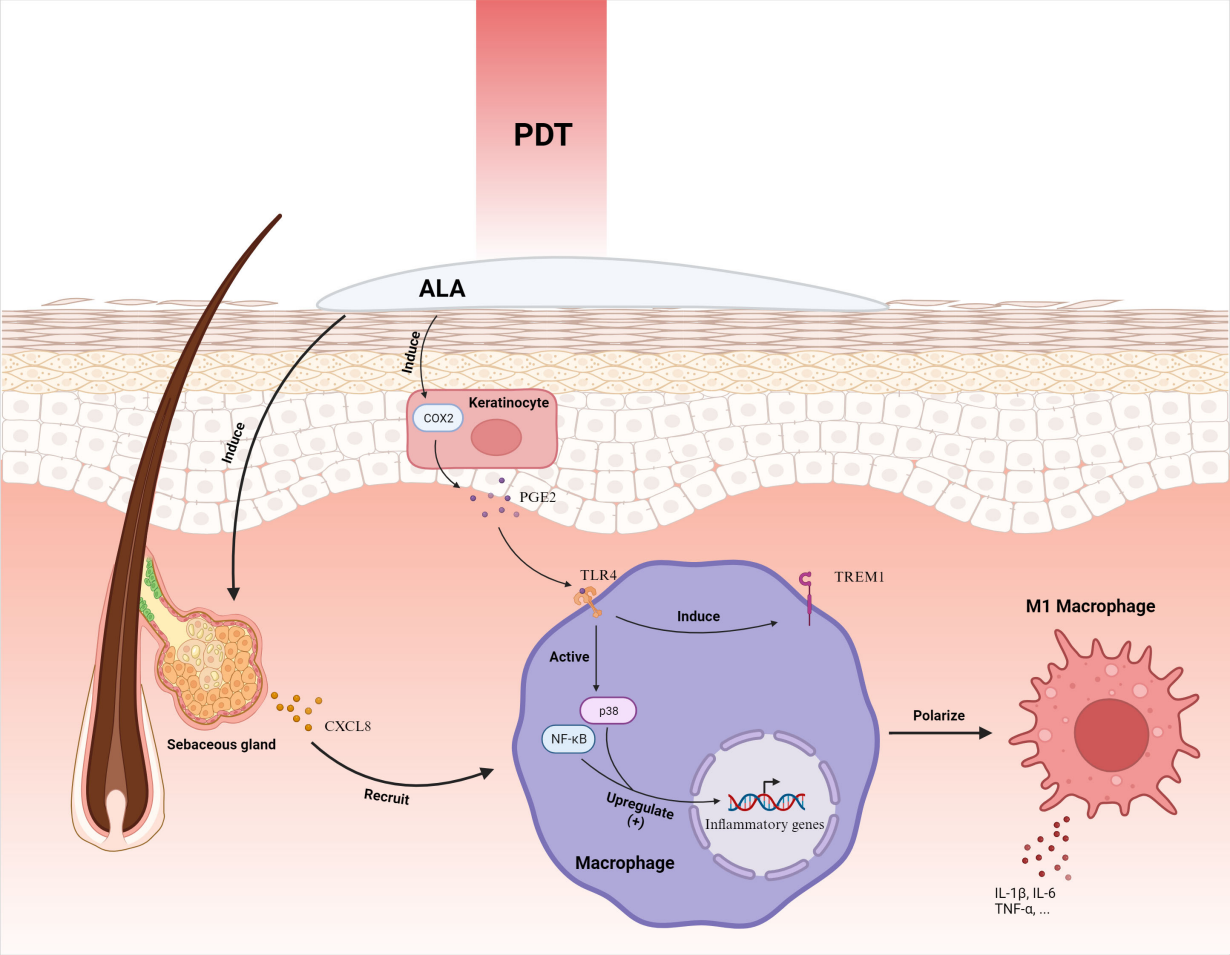
ALA-PDT orchestrates the mobilization of macrophages by inciting the secretion of CXCL8 from sebaceous gland cells. Furthermore, it intensifies the expression of COX2 in keratinocytes, culminating in the augmentation of PGE2. This molecule interacts with the TLR4 receptor on macrophages, instigating the activation of p38 and NF-κB pathways and ensuing in the elevation of inflammatory gene expression. Concurrently, it enhances the manifestation of TREM1 receptors, steering the polarization of macrophages toward the M1 phenotype, thereby amplifying the inflammatory response. ALA-PDT, Aminolevulinic Acid Photodynamic Therapy; COX2, Cyclooxygenase-2; CXCL8, Chemokine (C-X-C motif) ligand 8; IL-1β, Interleukin-1 beta; PGE2, Prostaglandin E2; TLR4, Toll-Like Receptor 4; TNF-α, Tumor Necrosis Factor-alpha; TREM1, Triggering Receptor Expressed on Myeloid cells 1. Adapted from “Skin (Layout)” by BioRender.com (2023). Retrieved from https://app.biorender.com/biorender-templates.

Zhang et al. delineated that ALA-PDT activates the p38 MAPK pathway, instigating CXCL8 expression in sebaceous gland cells and subsequently enlisting macrophages to augment the inflammatory response (75). Subsequently, Liu et al. elucidated that keratinocytes subjected to ALA-PDT exhibit escalated COX2 expression, releasing PGE2 to elevate TREM1 receptor expression via TLR4 in macrophages, fostering macrophage M1 polarization and triggering the p38 and NF-κB signaling pathways, thereby intensifying inflammation (62) (**Figure 4**). It’s hypothesized that by directing macrophage M1 polarization to enhance inflammation, ALA-PDT may offer a revolutionary solution for acne treatment.

### 5.4 Traditional Chinese medicine interventions

Auriculotherapy, an integral component of Traditional Chinese Medicine (TCM), has been revered as a therapeutic intervention for conditions like inflammation, pain, and drug addiction. Zuo et al. executed auriculotherapy interventions, which encompass auricular bloodletting therapy (ABT), auricular point sticking (APS), and their combination (ABPS), on a rat model representing acne (76). The results unveiled that ABT, APS, and ABPS were proficient in prompting the initiation of M2-type macrophages via meticulous modulation of macrophage polarization. This resulted in the alleviation of acne symptoms and a reduction in serum levels of TNF-α and IL-1β in rats. Notably, APS also exhibited a significant reduction in the expression of the TLR2/NF-κB signaling pathway. The discoveries from this study reinforce the mechanistic underpinning of TCM auricular acupoint therapy in addressing inflammatory conditions like acne, adding substantial validation to the application of auriculotherapy in unconventional acne treatments.

### 5.5 Exploration of alternative therapeutic strategies

#### 5.5.1 Antimicrobial peptides

Antimicrobial peptides, inherent immune molecules in organisms, exhibit versatile antimicrobial properties. Research by Wu et al. elucidated that Cath-MH, originating from frog skin, exhibits potent antimicrobial efficacy against *C. acnes* by inhibiting the proliferation of *C. acnes* and curtailing inflammatory cytokine production (77). This is facilitated through the suppression of macrophage TLR expression via PAMP binding, highlighting antimicrobial peptides as potent candidates for acne interventions.

#### 5.5.2 Organic compounds

Several organic compounds have unveiled promising therapeutic efficacies for acne through macrophage modulation. A study by Shin et al. depicted the suppressive role of pyrrolidine dithiocarbamate (PDTC) on multiple inflammatory cytokines, achieved by inhibiting NF-κB signaling and NLRP3 activation in macrophages under the influence of *C. acnes* (78). Additionally, investigations by Kim et al. concluded that the benzoxathiolone BOT-64 could deter the synthesis of inflammatory mediators by blocking the NF-κB signaling pathway through the inhibition of IKKβ in macrophages (79). Concurrently, research by Chung et al. demonstrated the capacity of LYR-71, another benzoxathiolone, to inhibit IFN-γ-induced inflammatory responses, accomplished by uncoupling the tyrosine phosphorylation of STAT-1 in macrophages (80).

#### 5.5.3 Additional therapeutic strategies

Non-steroidal anti-inflammatory drugs (NSAIDs) offer significant promise in acne treatment through novel mechanisms. Specifically, a study by Yang et al. substantiated that kinofen has the potential to inhibit NLRP3 inflammasome activation and reduce IL-1β expression, suggesting its applicability in treating acne vulgaris (81). Furthermore, innovative approaches such as microcurrent stimulation (MC) have been proposed by Lee et al., capable of mitigating inflammatory responses by modulating protein expression levels involved in TLR2/NF-κB signaling within macrophages (82). This highlights the potential of MC as a novel acne treatment method. Additionally, the role of probiotics in skin homeostasis and inflammation regulation cannot be overlooked. Wang et al. demonstrated that Bifidobacterium fermentum lysate (BFL) effectively mitigates LPS-induced secretion of IL-8, TNF-α, and COX-2 expression in macrophages (83). Collectively, these studies underscore the diverse and promising therapeutic strategies for acne treatment, ranging from NSAIDs and microcurrent stimulation to probiotics, each contributing uniquely to the understanding and management of acne (**Table 2**).

**Table 2:** Supplementary approaches for acne vulgaris focused on macrophage involvement.

Category	Chemical	Description	Mechanism of action	Source	References
Antimicrobial peptide	Cath-MH	Antimicrobial peptide from the skin of the frog Microhyla heymonsivogt	Killing bacteria and downregulating TLR expression	Microhyla heymonsivogt	(77)
Organic Compounds	PDTC	Hiol compound	Inhibition of the inflammatory response through suppression of the NF-κB pathway and activation of the NLRP3 inflammasome	Pyrrolidine dithiocarbamate	(78)
Organic Compounds	BOT-64	The novel small-molecule benzoxathiole 6,6-dimethyl-2-(phenylimino)-6,7-dihydro-5H-benzo-[1,3]oxathiol-4-one	Inhibition of inflammatory responses through suppression of the NF-κB pathway	Benzoxathiole derivatives	(79)
Organic Compounds	LYR-71	6-methyl-2-propylimino-6,7-dihydro-5H-benzo[1,3]oxathiol-4-one	Restraining IFN-gamma-induced inflammatory responses through uncoupling the tyrosine phosphorylation of STAT-1	Benzoxathiolone derivatives	(80)
NSAIDs	Auranofin	Anti-Rheumatic Gold Compound	Inhibition of inflammatory responses through suppression of NLRP3 inflammasome activation	/	(81)
Micro-current stimulation	MC	/	Inhibition of inflammatory responses through suppression of the TLR2/NF-κB pathway	/	(82)
Probiotic	BFL	Bifida Ferment Lysate	Reduction in the secretion of IL-8 and TNF-α, as well as downregulation of COX-2 expression	Bifidobacterium	(83)

## 6 Concluding remarks and perspectives

**Figure 5:**
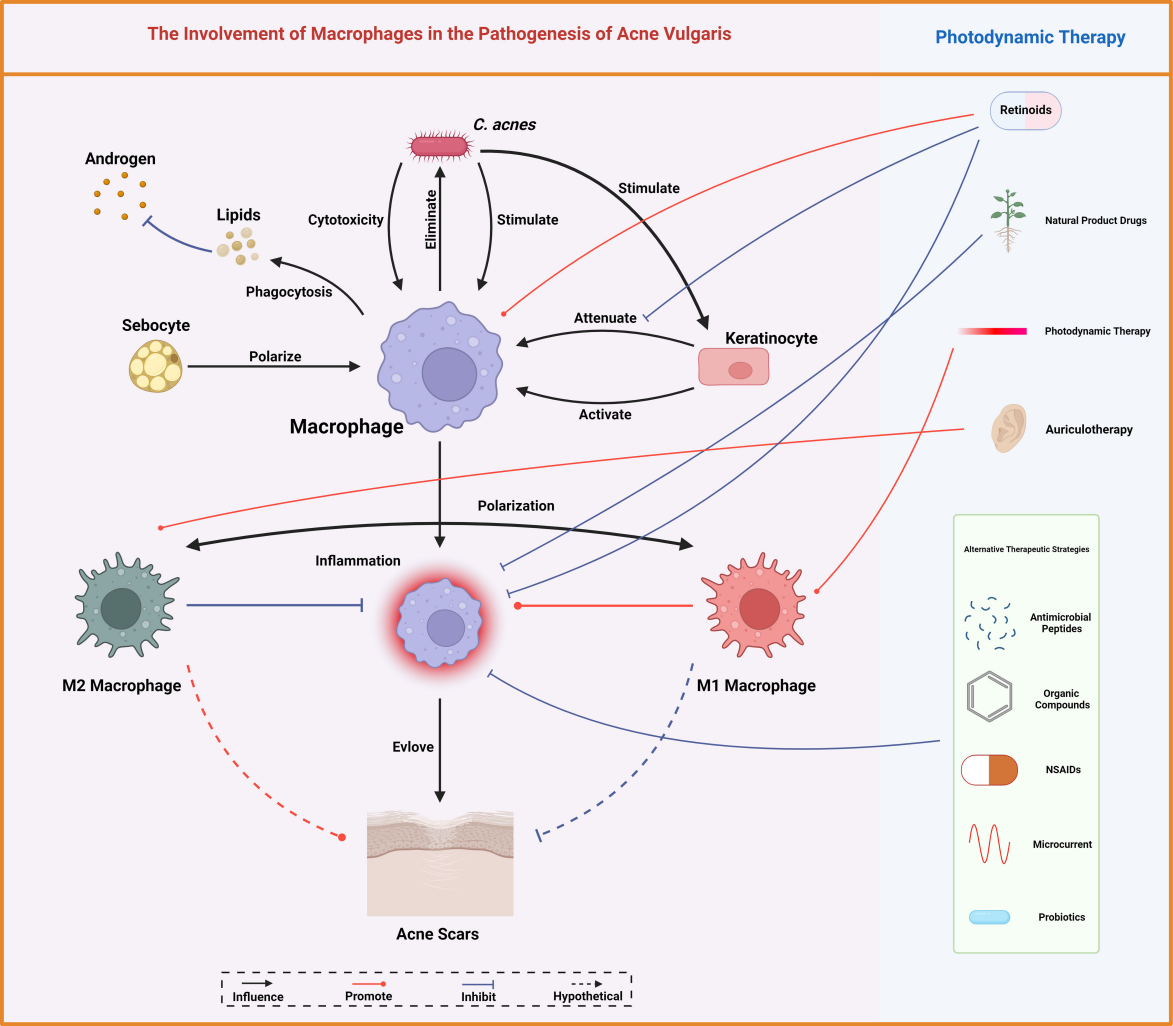
Macrophages mediate lipid homeostasis, bacterial eradication, immune modulation, and scar tissue formation, central to acne vulgaris pathobiology. Conventional therapeutic measures for acne vulgaris like retinoids and photodynamic therapy, alongside various other interventions, modulate these macrophage functions, offering therapeutic leverage against acne. Created with BioRender.com.

Macrophages indeed exemplify a double-edged sword in the pathogenesis of acne vulgaris. They are fundamental for sustaining homeostasis, modulating lipid, and neutralizing pathogens. Nevertheless, the overactivation of macrophages, underscored by the proinflammatory responses to *C. acnes*, can augment the severity of acne symptoms (**Figure 5**). The intricate mechanisms are being meticulously unraveled, albeit the methodology through which ALA-PDT modulates macrophage polarization and escalates the inflammatory response remains obscure. This ambiguity may be attributed to the advanced extermination of bacteria and the attenuation of scarring, necessitating more rigorous studies for verification. Given the pivotal role of macrophage polarization in the pathogenesis of myriad skin diseases, the imperative for intensified exploration of its implications in acne vulgaris is underscored (84).

In addressing acne, therapeutic interventions necessitate a comprehensive consideration of immune responses, anti-inflammatory, and antimicrobial approaches. Probing the alterations in macrophage activity in acne vulgaris promises enhanced comprehension of this prevalent dermatological ailment’s pathogenesis. Current interventions primarily employ anti-inflammatory strategies, with emerging evidence supporting the feasibility of macrophage modulation as a potential therapeutic avenue. The unveiling of novel therapeutic pathways, such as the employment of statins to inhibit mevalonate, is underway (85). Nevertheless, the pro-inflammatory properties of ALA-PDT pose significant challenges to existing anti-inflammatory paradigms. This brings forth pivotal queries: Should we consider the implementation of M1 macrophage polarization in patients manifesting suitable conditions (86)? Additionally, can ALA-PDT therapy be administered in patients with mild conditions to halt disease progression, serving as an expansion of the existing indications for ALA-PDT (87)? Such inquiries indubitably mandate more extensive and focused research endeavors into the pathogenesis and treatment methodologies of acne vulgaris.

## Author contributions

YF: Writing – original draft. JL: Writing – original draft. XM: Writing – review & editing. QJ: Writing – review & editing.
